# Divergent pandemic responses in miscarriage and ectopic pregnancy admissions—authors’ reply

**DOI:** 10.1016/j.lanepe.2026.101880

**Published:** 2026-09-22

**Authors:** Sindhu Sekar, Joshua Odendaal, Siobhan Quenby, Nicola Tempest

**Affiliations:** a Department of Women's and Children's Health, Centre for Women's Health Research, Institute of Life Course and Medical Sciences, University of Liverpool, Liverpool Health Partners, Liverpool, L8 7SS, UK; b Hewitt Centre for Reproductive Medicine, Liverpool Women's NHS Foundation Trust, Liverpool, L8 7SS, UK; c Liverpool Women's NHS Foundation Trust, Liverpool Health Partners, Liverpool, L8 7SS, UK; d NIHR Applied Research Collaboration Northwest Coast, University of Liverpool, Liverpool, UK; e Tommy's National Centre for Miscarriage Research, University Hospitals Coventry and Warwickshire NHS Trust, Coventry, CV2 2DX, UK; f Warwick Medical School, University of Warwick, Coventry, CV4 7AL, UK

We thank Cockburn and colleagues for their thoughtful correspondence and their interpretation of the findings.^1^ Admissions began to rise gradually in 2022 and 2023, alongside restoration of services from mid-2022,^2^ making changes in access and care-seeking plausible contributors. Nevertheless, changes in established and emerging risk factors for miscarriage remain relevant and cannot be disentangled from service effects using admission data alone.

The ectopic pregnancy comparison requires similar caution. The rise in population incidence reported between 2006 and 2021^3^ does not map directly onto hospital admissions. Over the same period, increasing use of expectant and outpatient medical management has progressively uncoupled diagnosis from admission, plausibly contributing to the downward trend and subsequent plateau in admissions despite rising incidence. The absence of a pandemic fall is also informative: ectopic pregnancy remained an urgent condition requiring assessment, while uncertainty around COVID-19 and the theoretical immunosuppressive effect of methotrexate might have altered the balance between medical and surgical management.^4^

For both conditions, admission trends reflect not only their underlying risk factors, but also when, where, and how early pregnancy care is delivered.

## Contributors

S.S., O.J., S.Q., and N.T. contributed to writing-original draft and writing review and editing.

S.S., O.J., S.Q., and N.T. were responsible for the decision to submit the reply for publication.

## Declaration of interests

All authors declare no competing interests.

## References

[1] Cockburn N., Chandan J.S., Parry-Smith W. (2026). Divergent pandemic responses in miscarriage and ectopic pregnancy admissions. Lancet Reg Health Eur.

[2] Coronavirus » next steps on transitioning from COVID-19 response to recovery. https://www.england.nhs.uk/coronavirus/next-steps-on-transitioning-from-covid-19-response-to-recovery/.

[3] Cockburn N., Hammond B., Gani I. (2024). Automating incidence and prevalence analysis in open cohorts. BMC Med Res Methodol.

[4] Royal College of Obstetricians and Gynaecologists (2021). Guidance for rationalising early pregnancy services in the evolving coronavirus (COVID-19) pandemic: Information for healthcare professionals (Version 2). RCOG. https://www.rcog.org.uk/guidance/coronavirus-covid-19-pregnancy-and-women-s-health/archived-guidance/.

